# Data-driven 1D design model for monotonic lateral loading of monopile foundations

**DOI:** 10.1016/j.mex.2025.103738

**Published:** 2025-11-26

**Authors:** Ioannis Kamas, Stephen K. Suryasentana, Harvey J. Burd, Byron W. Byrne

**Affiliations:** a Department of Engineering Science, University of Oxford, Oxford, UK; b Department of Civil and Environmental Engineering, University of Strathclyde, Glasgow, UK

**Keywords:** Monopile design, PISA, Soil-structure interaction, Offshore geotechnical engineering, Machine learning

## Abstract

Monopiles are a widely-used foundation system for offshore wind turbine support structures. In current practice, design calculations typically employ one-dimensional (1D) models in which the monopile is represented as an embedded beam. The current study presents a data-driven 1D design model for the analysis of offshore monopiles subjected to monotonic lateral load and moment loading. The method is based on the PISA design model framework; enhancements are incorporated in the model to improve its accuracy, scalability and to facilitate applications to a wide range of geotechnical conditions. The data-driven model incorporates a spline-based parametrisation of the soil reaction curves combined with machine learning techniques. The model is calibrated using a database of previously-published three-dimensional finite element calibration analyses. The method described in the current paper is concerned with:

• Modifications to the PISA design model framework to develop a data-driven 1D design model.
• Calibration of the data-driven 1D model for ground conditions comprising: (i) offshore glacial tills with varying strength–stiffness properties, and (ii) sands with a wide range of relative densities.
• Validation of the proposed method by comparing 1D model predictions for monopiles in homogeneous and layered soils with detailed 3D finite element analyses.

## Graphical abstract

**Figure:**
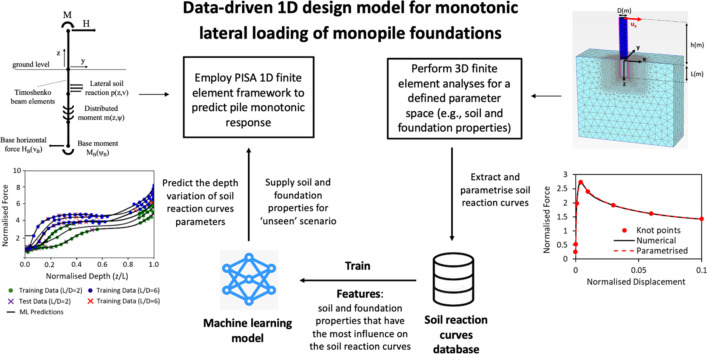

**Specifications table**

**Table:** 

**Subject area**	Engineering
**More specific subject area**	Offshore geotechnical engineering
**Name of your method**	Data-driven 1D design model for monotonic lateral loading of monopile foundations
**Name and reference of original method**	PISA Design Model Burd, H.J., Taborda, D.M.G. Zdravković, L., Abadie, C.N., Byrne, B.W., Houlsby, G.T., Gavin, K.G., Igoe, D.J.P., Jardine, R.J., Martin, C.M., McAdam, R.A., Pedro, A.M.G., Potts, D.M., 2020a. PISA design model for monopiles for offshore wind turbines: application to a marine sand. *Geotechniqu*e, 70(11), 1048–1066. Burd, H.J., Abadie, C.N., Byrne, B.W., Houlsby, G.T., Martin, C.M., McAdam, R.A., Jardine, R.J., Pedro, A.M., Potts, D.M., Taborda, D.M. and Zdravković, L., 2020b Application of the PISA design model to monopiles embedded in layered soils. *Géotechnique*, 70(11), pp.1067–1082. Byrne, B.W., Houlsby, G.T., Burd, H.J., Gavin, K.G., Igoe, D.J.P, Jardine, R.J., Martin, C.M., McAdam, R.A., Potts, D.M., Taborda, D.M.G., Zdravković, L., 2020. PISA design model for monopiles for offshore wind turbines: application to a stiff glacial clay till. *Geotechnique* 70(11), 1030–1047.
**Resource availability**	*Data will be made available on request*

## Background

Simplified models based on one-dimensional (1D) representations of embedded monopiles are widely used in the design of offshore wind turbine foundations. The traditional *p–y* method (e.g., [11,20,24]), which models the pile as an elastic beam and uses empirically derived *p–y* curves to represent soil resistance, has long served as the industry standard. However, design guidance implementing this method (e.g., [1]) was developed primarily for slender piles and may be unreliable for large-diameter monopiles with low length-to-diameter ratios, commonly used in offshore wind applications (e.g. [7,13]).

To address these limitations, the PISA (Pile Soil Analysis) project [8] developed an improved 1D design model for laterally loaded monopiles. The PISA design model builds on the *p–y* framework but incorporates two key innovations: (i) the use of four separate soil reaction components (distributed lateral load, distributed moment, base shear, and base moment) rather than a single *p–y* curve, and (ii) direct calibration from 3D finite element (FE) analyses. This calibration process allows the model to adapt to site-specific conditions by leveraging site investigation data in conjunction with 3D finite element simulations. PISA design model calibrations are therefore not static – they can develop in response to ongoing improvements in site characterisation and geotechnical analysis procedures. In this way, the PISA design model functions as a surrogate model – a computationally efficient approximation of more expensive 3D finite element simulations – supporting rapid yet robust design calculations.

The present study builds on the PISA design model concept by developing a more generalised, data-driven 1D design framework. The model retains the same underlying 1D finite element structure of the PISA design model — employing Timoshenko beam theory to represent the monopile and consistent 1D elements to represent the distributed soil reactions. However, instead of using fixed empirical functions, the soil reaction curves (i.e. relationships between the local rotation or displacement and the corresponding soil reaction component) are constructed using machine learning (ML) regression techniques. This development allows for more accurate representation of complex, nonlinear soil behaviour while maintaining computational efficiency suitable for design workflows. Furthermore, the PISA design approach is inherently limited in its ability to generalise to a wide range of geotechnical conditions due to its simplified curve-fitting calibration procedure. Its parameters are obtained through manual or semi-empirical fitting, which restricts systematic extension to more complex soil conditions. In contrast, the current design model employs a formal machine learning framework to overcome these limitations.

The proposed data-driven framework employs Gaussian Process Regression (GPR) to define the soil reaction curves. GPR offers high predictive accuracy even with limited training datasets and provides inherent uncertainty quantification. Unlike the PISA design model – which employs compact, human-readable parameter lists – the data-driven approach handles calibration data entirely within the ML framework. This offers greater flexibility in how data are structured, updated, and scaled. By removing artificial constraints on model formulation, the framework is capable of adapting to more complex geotechnical conditions and can scale as additional data become available.

## Method details

The data-driven 1D design framework is demonstrated using two benchmark calibration spaces established in the PISA project: Cowden till, a stiff glacial clay [9], which was later extended by Kamas et al. [16] to encompass a broader range of glacial till profiles with varying strength and stiffness properties; and Dunkirk sand, a marine sand [5] representing a range of relative densities. The same 3D finite element datasets that were previously used to calibrate and validate the 1D models in Burd et al. [5] and Kamas et al. [16] are employed here for both training and validation. Although this paper focuses on a well-established and limited calibration space, the data-driven framework is inherently scalable. It can be extended to more complex scenarios – including direct calibration for layered soils incorporating the geometry of the layers as features (e.g. [18]) and geotechnical materials exhibiting complex behaviour (e.g. chalk) – by incorporating additional geotechnical features into the learning process. These extensions have been developed by the authors and will be presented in future publications.

**Fig. 1:**
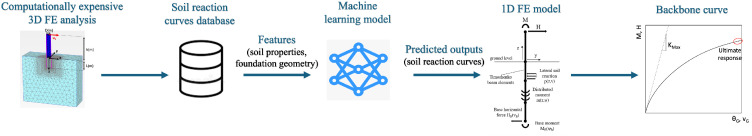
Schematic flowchart indicating the key stages to develop a data-driven 1D design model for lateral loaded monopile foundations.

This section outlines the full methodology, beginning with the extraction, normalisation, and parametrisation of soil reaction curves, followed by feature selection, GPR machine learning model training, and the application of a 1D finite element framework to predict the monotonic lateral response of the pile. Fig. 1 illustrates a schematic flowchart showing how the proposed data-driven model works for generating a new 1D model.

### Soil reaction curves representation in the data-driven 1D design model

**Fig. 2:**
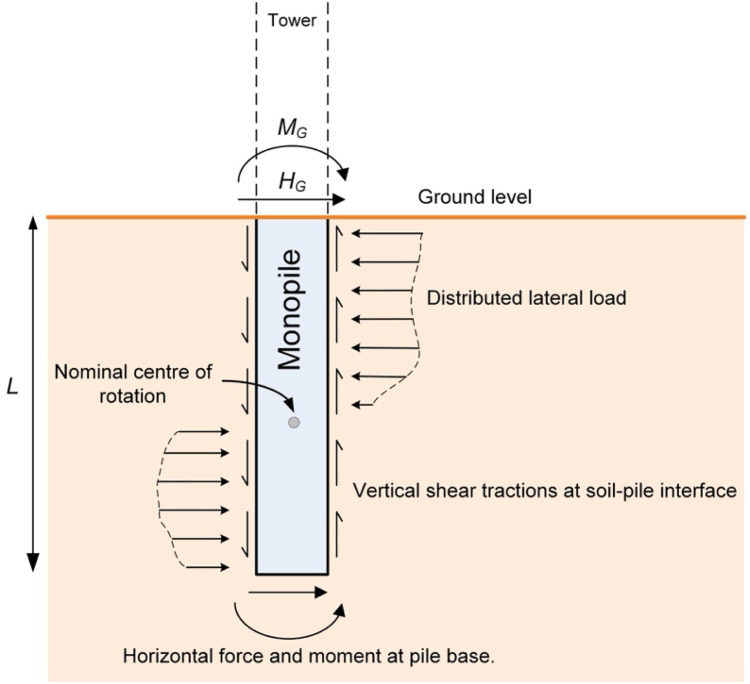
Soil reaction components of the PISA design model (adapted from [9]). The tractions shown are those assumed to act on the embedded pile.

Consistent with the PISA design model, the calibration procedure for the data-driven 1D design model requires that 3D finite element calibration analyses are conducted across a predefined calibration space. Tractions along the soil-pile interface are extracted from these analyses and used to derive numerical soil reaction curves corresponding to four separate components: distributed lateral load, distributed moment, base shear, and base moment, as described by Byrne et al. [9] and Burd et al. [5] and illustrated in Fig. 2.

### Soil reaction curves normalisation

The PISA design model adopts a normalisation approach in which the relevant displacement and rotation variables – and the corresponding load and moment variables – are scaled so that the soil reaction curves can be expressed as functions of a single dimensionless variable. The normalised variables employed in this approach are listed in Table 1. The underlying assumption in the PISA design model is that, when expressed in dimensionless form, the (normalised) soil reaction curves at each location on the monopile collapse onto a function of a single dimensionless variable. Experience from the application of the PISA design model [5,9] suggests that this approach is generally robust. However, numerical soil reaction curves extracted from 3D finite element analyses do not universally conform to a unique normalised response. In particular, the shape of the soil reaction curves for clay is found to depend on the shear modulus ratio $G_{0}/s_{u}$; this dependency is not captured when the PISA normalisations are adopted. Additionally, the parameters required to fit the soil reaction curves (employing a four-parameter conic function) to the numerical soil reaction curves extracted from 3D finite element analysis for distributed lateral load and moment depend to some extent on the pile length-to-diameter ratio L/D; but the PISA framework does not explicitly allow this dependency to be accounted for.

**Table 1:** Dimensionless forms adopted in the current data-driven model. Also shown for comparison are the dimensionless forms adopted in the PISA design model [5,9]. The ‘clay framework’ is employed in the Cowden till model; the ‘sand framework’ is employed in the Dunkirk sand model. Values of undrained shear strength $s_{u}$ correspond to measurements in triaxial compression; $\sigma_{vi}^{\prime }$ is the local value of the initial vertical effective stress in the soil.

Normalised variable	Clay framework		Sand framework	
	Data-driven model	PISA design model	Data-driven model	PISA design model
Distributed lateral load, $\bar{p}$	$\frac{p}{s_{u}D}$	$\frac{p}{s_{u}D}$	$\frac{p}{\sigma_{vi\prime }D}$	$\frac{p}{\sigma_{vi\prime }D}$
Lateral displacement, $\bar{v}$	$\frac{v}{D}$	$\frac{vG_{0}}{Ds_{u}}$	$\frac{v}{D}$	$\frac{vG_{0}}{D\sigma_{vi}^{\prime }}$
Distributed moment, $\bar{m}$	$\frac{m}{s_{u}D^{2}}$	$\frac{m}{s_{u}D^{2}}$	$\frac{m}{\sigma_{vi}^{\prime }D^{2}}$	$\frac{m}{|p|D}$
Pile cross-section rotation, $\bar{\psi }$	ψ	$\frac{\psi G_{0}}{s_{u}}$	ψ	$\frac{\psi G_{0}}{\sigma_{vi}^{\prime }}$
Base horizontal force, ${\bar{H}}_{B}$	$\frac{H_{B}}{s_{u}D^{2}}$	$\frac{H_{B}}{s_{u}D^{2}}$	$\frac{H_{B}}{\sigma_{vi}^{\prime }D^{2}}$	$\frac{H_{B}}{\sigma_{vi}^{\prime }D^{2}}$
Base moment, ${\bar{M}}_{B}$	$\frac{M_{B}}{s_{u}D^{3}}$	$\frac{M_{B}}{s_{u}D^{3}}$	$\frac{M_{B}}{\sigma_{vi}^{\prime }D^{3}}$	$\frac{M_{B}}{\sigma_{vi}^{\prime }D^{3}}$

In the current data-driven 1D design model, numerical soil reaction curves (i.e. corresponding to the data extracted from the 3D finite element calibration analyses) are initially scaled using the dimensionless variables listed in the ‘Data-driven model’ columns of Table 1. Notably, the normalisation of the displacement and rotation variables omits the shear modulus ratios $G_{0}/s_{u}$ and $G_{0}/\sigma_{vi}^{\prime }$ that appear in the PISA normalisation; instead, these ratios appear as input features elsewhere in the model. This modification allows the model to learn any nonlinear relationships involving stiffness independently of the normalisation process. For sand, the moment normalisation $\bar{m}=m/\left(\sigma_{vi}^{\prime }D^{2}\right)$ is adopted in place of the corresponding formulation in the PISA model, which links the distributed lateral load (p) to the distributed moment soil reaction component (m). This change was introduced to avoid numerical implementation issues encountered with the PISA distributed moment scaling, while retaining physical consistency with the underlying geotechnical parameters.

### Soil reaction curve parametrisation

The PISA design model employs a relatively simple conic function to represent the soil reaction curves. This function is defined by only four parameters, the values of which – and their variation with depth – can be conveniently tabulated (e.g., Table 4 in [9]; Table 5 in [5]). However, this four-parameter formulation is not sufficient to capture the high degree of nonlinearity typically present in numerical soil reaction curves, as derived from traction data at the soil-pile interface in 3D finite element calibration analyses. The limited fidelity of the conic function limits the model’s ability to accurately replicate the behaviour observed in detailed simulations. As discussed in Byrne et al. [9] and Burd et al. [5], the performance of the PISA design model can be significantly improved through a ‘second-stage’ optimisation, in which the parameters of the soil reaction curves are adjusted to enhance the global response of the model. This need for additional optimisation is a consequence of the conic function’s limited ability to reproduce the true shape of the numerical soil reaction curves.

In the current work, this limitation is removed by replacing the four-parameter formulation with a more detailed spline-based representation of the soil reaction curves. While the spline representation requires a significantly larger set of calibration data, these data are efficiently managed using machine learning techniques. As a result, the soil reaction curves can be modelled at a higher level of fidelity, rendering the second-stage optimisation step – previously essential in the PISA model – unnecessary. In the data-driven 1D design framework normalised numerical soil reaction curves are represented as spline functions of the form $\bar{y}=g\left(\bar{u}\right)$ where $\bar{u}$ is a normalised displacement (or rotation) variable and $\bar{y}$ is the corresponding normalised load (or moment) variable as defined in the ‘Data-driven model’ columns in Table 1. Spline functions provide a convenient approach. For example, post-peak softening behaviour occasionally occurs in the soil reaction curves; splines provide a consistent way of representing this kind of behaviour. A Piecewise Cubic Hermite Interpolating Polynomial (PCHIP) interpolation scheme [14] is employed to define the spline. The PCHIP scheme is adopted, rather than a more conventional cubic spline interpolation, as it avoids spurious oscillations and can accurately connect flat regions (which often occur in numerical soil reaction curves). The PCHIP algorithm achieves this by defining the slopes at the knot points such that the interpolating polynomial respects the monotonicity of the data [14] – if the data exhibit non-monotonic behaviour with linear gradients $\delta_{k-1}$ and $\delta_{k}$ of opposite signs, indicating softening, the slope calculated by the PCHIP algorithm between those points is zero.

**Fig. 3:**
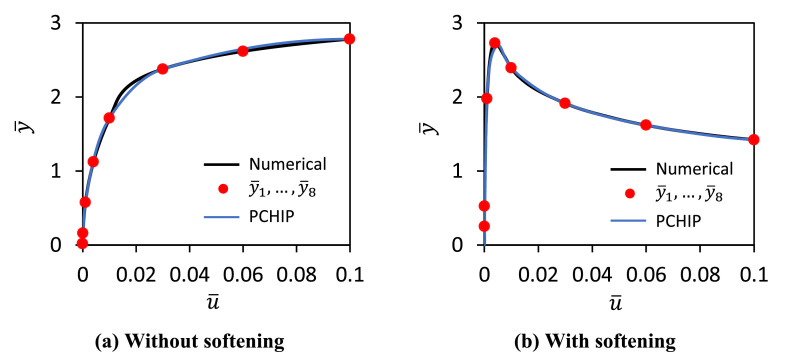
Example forms of the eight knot point PCHIP soil reaction curves: (a) without softening, (b) with softening. The ‘Numerical’ data relate to normalised numerical soil reaction curves; ‘PCHIP’ refers to the spline curve that is defined by the knot points shown as red filled circles.

An approach is adopted in which eight knot points are used to define each PCHIP spline; example cases employing eight knot points are shown in Fig. 3. Pre-defined values of normalised displacement $\bar{u}$ at the knot points are adopted as indicated in Table 2; this distribution was selected to provide appropriate resolution for both small and large displacements/rotations. Values of normalised load or moment, ${\bar{y}}_{i}$, at each knot point are determined directly from the individual normalised numerical soil reaction curves. Some of the numerical distributed lateral load soil reactions near the pile centre of rotation (see Fig. 2) are not fully mobilised up to $\bar{u}=0.1$ in the 3D finite element calibration calculations. In these cases, extrapolation is applied by setting the force/moment value equal to the last calculated numerical data. Examples of PCHIP interpolated soil reaction curves are shown in Fig. 3; these plots illustrate the ability of the formulation to capture hardening and softening behaviour.

**Table 2:** Knot point data for eight-point PCHIP splines. The values of $\bar{u}$ are pre-defined in the model. Values of $\bar{y}$ are determined to provide a fit with the numerical soil reaction curves.

$\bar{u}$	0	0.00004	0.0001	0.001	0.004	0.01	0.03	0.06	0.1
$\bar{y}$	0	${\bar{y}}_{1}$	${\bar{y}}_{2}$	${\bar{y}}_{3}$	${\bar{y}}_{4}$	${\bar{y}}_{5}$	${\bar{y}}_{6}$	${\bar{y}}_{7}$	${\bar{y}}_{8}$

**Fig. 4:**
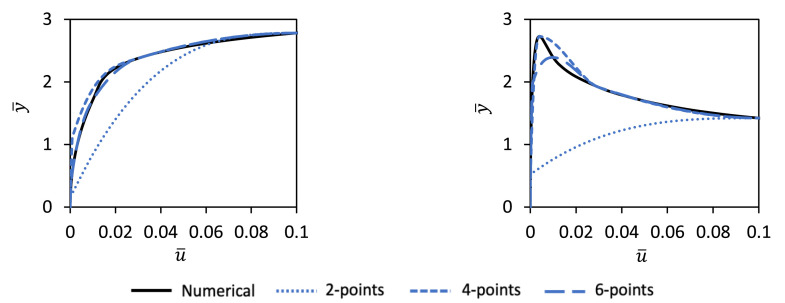
Performance of PCHIP spline fits with different numbers of knot points.

The use of eight knot points for the spline representation was chosen via experimentation as a suitable balance between model complexity and predictive accuracy. However, it should be noted that the current method could be employed with splines with a different number of knot points. Fig. 4 illustrates an example investigation examining how varying the number of knot point influences the fidelity of the spline representation of the soil reaction curves. It is clear from this figure that: (i) an eight knot point spine provides a close representation of the numerical soil reaction curves and (ii) the fidelity of the spline representation reduces significantly as the number of knot points is reduced.

### Specification of calibration data

The data-driven 1D design model developed in this study can be calibrated for arbitrary pile and soil configurations. However, to demonstrate the methodology, the present work focuses on the calibration spaces originally used in the development of the PISA design model – i.e. the general Dunkirk sand model [5] and the general Cowden till model which is based on the Cowden till properties defined by Byrne et al. [9] with extensions by Kamas et al. [16] for a broader range of clay strength and stiffness properties. The training data employed in the current study are sourced from existing databases of 3D finite element analyses. These simulations were performed using the ICFEP finite element software [23] for the general sand model (as in [5]), and the PLAXIS 3D software [4] for the general clay model (as described in [16]). The calibration space and the associated 3D finite element analyses are briefly summarised below; for full details, the reader is referred to the original sources.

**Fig. 5:**
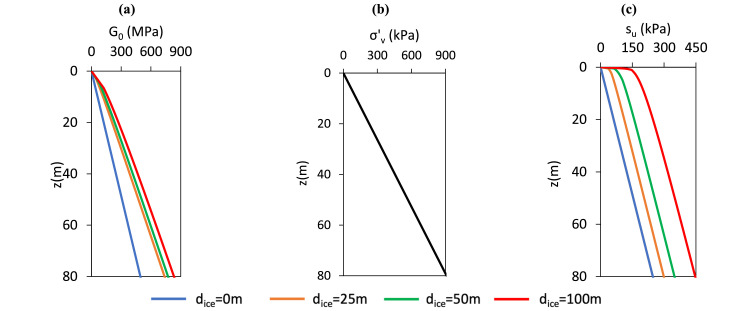
Depth profiles of $G_{0}$, $\sigma {}_{v}^{\prime }$,$\;s_{u}$ for the representative offshore glacial till sites employed in the current model. The vertical effective stress profile is identical across all glacial till sites.

**Fig. 6:**
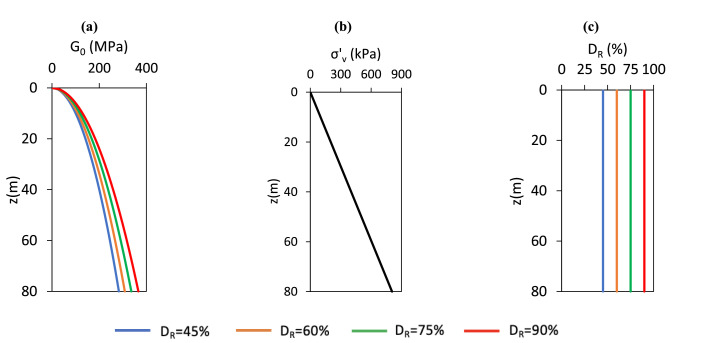
Depth profiles of $G_{0}$, $\sigma {}_{v}^{\prime }$, $D_{R}$ for the representative offshore sand sites employed in the current model. The vertical effective stress profile is identical across all sand sites.

Two soil types are considered: Cowden till and Dunkirk sand**.** The Cowden till model is based on the ‘representative offshore glacial till’ framework described by Kamas et al. [16]. This model which incorporates soils with a range of consolidation histories in which overconsolidation is assumed to result solely from the historical action of ice sheet loading. Specifically, the preconsolidation stress is estimated by applying an overburden pressure equivalent to the weight of a hypothetical ice sheet of thickness $d_{ice}$, acting on the current ground surface. The Dunkirk sand model follows the ‘representative offshore homogeneous sand’ calibration space developed in Burd et al. [5], incorporating sand profiles with relative densities ($D_{R}$) of 45 %, 60 %, 75 %, and 90 %. Profiles of the relevant soil parameters for the representative clay and sand sites are illustrated in Fig. 5, Fig. 6. The pile configurations adopted in the calibration analyses, which are the same as those used in the original PISA model development [9], are listed in Table 3.

**Table 3:** Pile geometry calibration space. D is pile diameter; h is load eccentricity; L is pile embedded length; t is pile wall thickness. Data reproduced from Byrne et al. [9].

**Pile**	***D* (m)**	***H* (m)**	***h / D***	***L* (m)**	***L / D***	***t* (mm)**	***D / t***
C1	10	50	5	20	2	91	110
C2	10	150	15	20	2	91	110
C3	10	50	5	20	2	125	80
C4	10	50	5	60	6	91	110
C5	10	150	15	60	6	91	110
C6	5	25	5	10	2	83	60
C7	5	25	5	10	2	45	110
C8	5	25	5	30	6	45	110
C9	5	75	15	30	6	45	110
C10	7.5	37.5	5	15	2	68	110
C11	7.5	37.5	5	45	6	68	110

### Feature selection

The function $g(\bar{u}$) employed to represent the soil reaction curves in the data-driven model is a spline defined by eight knot points for which the value ${\bar{y}}_{i}$ at each of knot point is a function of the features $f_{j}=1,\ldots ,n$ that are selected to represent the problem, where n is the number of features being employed. The soil reaction curves are therefore implicit functions of the relevant displacement/rotation variable and the features as $\bar{y}=g\left(\bar{u}|f_{1},f_{2,\;\ldots },f_{n}\right)$. To develop this modelling approach, a suitable set of features needs to be selected.

A systematic procedure is adopted to select appropriate features as follows. The key geometric variables defining the problem are {L,D,t,h,z} where z is distance below ground level. These (five) variables may be grouped into a set of (four) dimensionless quantities$\;\{\frac{t}{D},\frac{h}{D},\frac{z}{L},\frac{L}{D}\}$ referred to as ‘synthetic features’. (Note the convention adopted in the current paper is that quantities written inside curly braces {■} are features or synthetic features). In this synthetic feature set the quantity $\left\{\frac{z}{L}\right\}$ is preferred to alternatives such $\left\{\frac{z}{D}\right\}$ to describe the depth variation of the soil reaction curve parameters as it has a defined range between 0 and 1 which is favourable for ML models. To identify the most expressive and non-redundant features for each knot point parameter $y_{i}$ associated with distributed load/moment and base force/moment soil reactions, multiple complementary techniques were employed. These included heatmap-based correlation matrices and pair plots to assess feature interdependencies, as well as Spearman correlation coefficients to evaluate monotonic relationships. In addition, formal feature selection was performed using multi-task LASSO regression (see LASSO model weights in Table 4, Table 5) with 5-fold cross-validation [21]. This method consistently indicated that the most relevant geometric features are $\left\{\frac{z}{L},\frac{L}{D}\right\}$ for the distributed load and moment soil reactions, and $\left\{\frac{L}{D}\right\}$ for the base reactions. These ‘geometric’ features were therefore selected for use in the final ML models.

**Table 4:** Values of weights (rounded to three decimal places) determined by multi-task LASSO regression for the knot point parameters associated with the representative offshore glacial clay till sites. The average mean squared error (MSE) for the LASSO regression is 0.025 for the $\bar{p}$*,*$\bar{m}$ knot points and 0.029 for the ${\bar{H}}_{B}$*,*${\bar{M}}_{B}$ knot points.

Component	${\bar{y}}_{1}$	${\bar{y}}_{2}$	${\bar{y}}_{3}$	${\bar{y}}_{4}$	${\bar{y}}_{5}$	${\bar{y}}_{6}$	${\bar{y}}_{7}$	${\bar{y}}_{8}$
$\bar{p}$	z/L=0.293	z/L=0.432	z/L=0.636	z/L=0.523	z/L=0.562	z/L=0.594	z/L=0.585	z/L=0.580
	L/D=-0.076	L/D=-0.048	L/D=0.046	L/D=0.140	L/D=0.176	L/D=0.210	L/D=0.210	L/D=0.213
	h/D=0	h/D=0	h/D=0	h/D=0	h/D=0	h/D=0	h/D=0	h/D=0
	t/D=0	t/D=0	t/D=0	t/D=0	t/D=0	t/D=0	t/D=0	t/D=0

$\bar{m}$	z/L= 0.118	z/L= 0.081	z/L= 0.246	z/L= 0.127	z/L= -0.043	z/L= -0.261	z/L= -0.217	z/L= -0.223
	L/D= 0.005	L/D= -0.143	L/D= -0.026	L/D= 0.128	L/D= 0.011	L/D= -0.103	L/D= -0.131	L/D= -0.144
	h/D=0	h/D=0	h/D=0	h/D=0	h/D=0	h/D=0	h/D=0	h/D=0
	t/D=0	t/D=0	t/D=0	t/D=0	t/D=0	t/D=0	t/D=0	t/D=0

${\bar{H}}_{B}$	L/D=-0.653	L/D=-0.434	L/D=0.550	L/D=0.741	L/D=0.767	L/D=0.629	L/D=0.610	L/D=0.634
	h/D=0	h/D=0	h/D=0	h/D=0	h/D=0	h/D=0	h/D=0	h/D=0
	t/D=0	t/D=0	t/D=0	t/D=0	t/D=0	t/D=0	t/D=0	t/D=0

${\bar{M}}_{B}$	L/D=0.109	L/D=0.511	L/D=-0.239	L/D=-0.544	L/D=-0.641	L/D=-0.762	L/D=-0.796	L/D=-0.780
	h/D=0	h/D=0	h/D=0	h/D=0	h/D=0	h/D=0	h/D=0	h/D=0
	t/D=0	t/D=0	t/D=0	t/D=0	t/D=0	t/D=0	t/D=0	t/D=0

**Table 5:** Values of weights (rounded to three decimal places) determined by multi-task LASSO regression for the knot point parameters associated with the PISA representative offshore homogenous sand sites. The average mean squared error (MSE) for the LASSO regression is 0.141 for the $\bar{p}$*,*$\bar{m}$ knot points and 0.017 for the ${\bar{H}}_{B}$*,*${\bar{M}}_{B}$ knot points.

Component	${\bar{y}}_{1}$	${\bar{y}}_{2}$	${\bar{y}}_{3}$	${\bar{y}}_{4}$	${\bar{y}}_{5}$	${\bar{y}}_{6}$	${\bar{y}}_{7}$	${\bar{y}}_{8}$
$\bar{p}$	z/L=-0.210	z/L=-0.194	z/L=-0.202	z/L=-0.223	z/L=-0.253	z/L=-0.268	z/L=-0.239	z/L=-0.173
	L/D=-0.105	L/D=-0.117	L/D=-0.091	L/D=-0.096	L/D=-0.111	L/D=-0.101	L/D=-0.053	L/D=0.050
	h/D=0	h/D=0	h/D=0	h/D=-0.001	h/D=-0.001	h/D=-0.001	h/D=-0.002	h/D=-0.002
	t/D=0	t/D=0	t/D=0	t/D=0	t/D=0	t/D=0	t/D=0	t/D=0

$\bar{m}$	z/L=-0.217	z/L=-0.232	z/L=-0.151	z/L=-0.195	z/L=-0.223	z/L=-0.232	z/L=-0.188	z/L=-0.154
	L/D=-0.074	L/D=-0.090	L/D=-0.049	L/D=-0.061	L/D=-0.069	L/D=-0.067	L/D=-0.046	L/D=-0.027
	h/D=0	h/D=0	h/D=0	h/D=0	h/D=0.001	h/D=0.001	h/D=-0.001	h/D=-0.002
	t/D=0	t/D=0	t/D=0	t/D=0	t/D=0	t/D=0	t/D=0	t/D=0

${\bar{H}}_{B}$	L/D=-0.623	L/D=-0.605	L/D=-0.543	L/D=-0.539	L/D=-0.435	L/D=-0.425	L/D=-0.534	L/D=-0.523
	h/D=-0.066	h/D=-0.056	h/D=-0.031	h/D=0.021	h/D=0.021	h/D=0.045	h/D=0.079	h/D=0.081
	t/D=0	t/D=0	t/D=0	t/D=0	t/D=0	t/D=0	t/D=0	t/D=0

${\bar{M}}_{B}$	L/D=-0.386	L/D=-0.398	L/D=-0.591	L/D=-0.655	L/D=-0.648	L/D=-0.708	L/D=-0.674	L/D=-0.626
	h/D=0.015	h/D=-0.052	h/D=-0.045	h/D=-0.015	h/D=-0.002	h/D=0.016	h/D=0.032	h/D=0.038
	t/D=0	t/D=0	t/D=0	t/D=0	t/D=0	t/D=0	t/D=0	t/D=0

Additional features need to be specified to represent the geotechnical conditions. In considering this issue a distinction needs to be made between ‘site-specific’ models (i.e. a calibration that is specific to a particular variation of soil strength and stiffness with depth) and ‘generalised’ models (in which the soil strength and stiffness profiles span a range of potential site conditions). In the current work, a generalised model is developed for Cowden till, encompassing a range of strength and stiffness profiles typical of glacial tills, and a corresponding generalised model is developed for Dunkirk sand for a range of relative densities. To represent soil stiffness, the features $\left\{\frac{G_{0}}{s_{u}}\right\}$ and $\left\{\frac{G_{0}}{\sigma_{vi}^{\prime }}\right\}$ are adopted for the clay and sand frameworks, respectively. These ‘geotechnical’ features are sufficient to describe soil stiffness in site-specific models. However, for generalised models – trained across a variety of soil conditions – additional ‘geotechnical’ features are required to account for the relative strength of the soil. For the generalised clay model, the feature $\left\{\frac{\sigma_{vi}^{\prime }}{s_{u}}\right\}$ is adopted; this ratio is closely related to the local overconsolidation ratio and thus provides a suitable measure of relative strength in cohesive soils. For the generalised sand model, the relative density $\left\{D_{R}\right\}$, is used to quantify relative strength. These additional features enable the model to accurately reflect varying soil conditions within a broad calibration space.

### Machine learning regression model

A key task for the data-driven model is to predict numerical values of the knot point data ${\bar{y}}_{i}$ for previously unseen values of the features (i.e. features not included in the training data). During the training process, training data comprising sets of data on $\left({\bar{y}}_{i},\;f_{j}\right)$ for i=1,…,8 and j=1,…,n are collected for all the soil reaction curves for all the calibration piles. For an example case of 11 calibration piles with 40 depth locations at which soil reaction curves are extracted from the calibration analysis (noting that each location contributes data for two curves corresponding to displacement and rotation), the total number of training data (i.e. the total number of knot points in the training data) are N=8×11×40×2=7040. This relatively large dataset can be efficiently handled using the machine learning methods described in the following sections.

To predict the knot point data ${\bar{y}}_{i}\;$for unseen cases a suitable ML regression model needs to be selected. In this study, Gaussian Process Regression (GPR) was employed. GPR demonstrated high predictive accuracy for the soil reaction curve data points and offers a key advantage in providing probabilistic predictions with associated uncertainty bounds. Therefore, GPR enables a quantifiable measure of confidence in the model’s predictions for previously unseen design cases, which is particularly valuable in engineering decision-making.

Moreover, GPR is highly data-efficient and well-suited to problems where training data may be limited – a common scenario when database generation requires time-consuming 3D finite element analyses. The use of Automatic Relevance Determination (ARD) kernels further enhances interpretability by identifying the most influential input features. Additionally, GPR produces continuous predictions that are consistent with the physical behaviour of the soil reaction curve knot-point parameters.

While alternative ML models – such as neural networks [15] and Extreme Gradient Boosting (XGBoost) [10] – also showed promise as regression candidates during preliminary evaluations, the data-driven 1D design models developed in the current study employ GPR.

### Gaussian process regression

Gaussian Process Regression (GPR) [25] employs a Gaussian probability distribution over random functions, updated using Bayes’ rule based on observed data. It is a non-parametric method, with model complexity growing with the size of the training dataset. It provides an automatic variance measure to quantify prediction uncertainty (from both the model and data noise). A GPR model is defined by its mean function m(x) and its covariance function (or kernel) $k(x,x^{\prime })$ where x refers to training data and $x^{\prime }$ to test (i.e. ‘unseen’) data.

GPR was implemented via the Scikit-learn Python library [22], adopting a zero-mean function m(x)=0. The Matérn kernel with ν=5/2 was employed, defined as:

(1) $$k_{Mat}\left(x,x^{\prime }\right)=\sigma_{f}^{2}\left(\frac{2^{1-\nu }}{\Gamma \left(\nu \right)}\right){\left(\frac{\sqrt{2\nu }}{l}\;d\left(x,x^{\prime }\right)\right)}^{\nu }K_{\nu }\left(\frac{\sqrt{2\nu }}{l}d\left(x,x^{\prime }\right)\right)$$

where Γ(ν) is the gamma function; $K_{v}$ is the modified Bessel function of the second kind; v is a positive parameter of the covariance that controls the smoothness of the function; $\sigma_{f}^{2}$ is the signal variance of the function; l is a characteristic length-scale; $d(x,x^{\prime })$ is the Euclidean distance between x and $x^{\prime }$.

Kernels define the similarity of two data points on the basis that similar features (i.e. inputs) should predict similar outputs (which in the current model are the soil reaction curve knot point data). The kernel’s hyperparameters, $\sigma_{f}^{2}$, $l_{i}$, are optimised by maximising the log marginal likelihood. Automatic Relevance Determination was employed to optimise these hyperparameters, allowing for different $l_{i}$ values for each feature, thereby enabling the model to weigh the relative importance of different inputs. To address potential noise in the data, a white noise kernel was added to the Matérn kernel, defined as:

(2) $$k_{WN}\left(x,\;x^{\prime }\right)=\left\{ \begin{array}{c} \sigma_{n}^{2}\;if\;x=x^{\prime }\;\\ 0\;otherwise \end{array}\right.$$

where $\sigma_{n}^{2}$ represents the noise variance. Model optimisation was performed using Scikit-learn default settings, with a convergence tolerance of $1\;\times 10^{-6}$ for tolerance and a maximum of 1000 iterations.

### Assessment of the GPR model

The total number of knot point data comprising the dataset used to train the GPR model for each soil reaction component is presented in Table 6. During the training process for each GPR model, the dataset corresponding to each knot point was randomly split, with 80 % allocated for training and 20 % reserved for testing ensuring that all configurations and soil conditions were represented. The training data were used to calibrate the model hyperparameters, while the test data evaluated each model's ability to generalise to unseen data. Completely separating data by individual pile geometries was not adopted, as this would require excluding entire finite element calibration analyses from the training set. Given that each 3D FE analysis requires considerable computational time and resources, such an approach would significantly reduce the available calibration data.

**Table 6:** Total number calibration data for each knot point for each soil reaction component.

Model name	$\bar{p}$	$\bar{m}$	${\bar{H}}_{B}$	${\bar{M}}_{B}$
Cowden Till	1482	1482	44	44
Dunkirk sand	1264	1264	44	44

To evaluate the performance of the GPR models, three metrics commonly used in regression analysis were employed: the coefficient of determination (*R*^2^), the mean squared error (MSE), and the mean absolute percentage error (MAPE).

(3) $$R^{2}=1-\frac{\sum_{i=1}^{n}{\left(y_{i}-{\hat{y}}_{i}\right)}^{2}}{\sum_{i=1}^{n}{\left(y_{i}-\bar{y}\right)}^{2}}$$

(4) $$MSE=\frac{1}{n}\sum_{i=1}^{n}{\left(y_{i}-{\hat{y}}_{i}\right)}^{2}$$

(5) $$MAPE=\frac{1}{n}\sum_{i=1}^{n}\left|\frac{y_{i}-{\hat{y}}_{i}}{y_{i}}\right|$$

where $y_{i}$ are the observed knot point data; (in the current context ‘observed’ corresponds to data from 3D finite element calibration analyses). ${\hat{y}}_{i}$ are the corresponding model predictions and $\bar{y}$ is the mean of the observed values. *R*^2^ quantifies the effectiveness of the model in capturing the variability in the observed data; its value typically ranges from 0 to 1. Higher *R*^2^ values indicate a better match between the observations and predictions. MSE measures the average of the squared differences between predicted and observed values, giving greater weight to larger deviations. MAPE represents the mean prediction error expressed as a percentage of the observed values.

Two additional metrics were used to assess the uncertainty of the GPR model predictions – the prediction interval coverage probability (PICP) and the mean prediction interval width (MPIW). These are defined as:

(6) $$PICP=\frac{1}{n}\sum_{i=1}^{n}1\left\{y_{i}\in \left[\mu_{i}+c\sigma_{i},\;\mu_{i}-c\sigma_{i}\right]\right\}\times \;100\%$$

(7) $$MPIW=\frac{1}{n}\sum_{i=1}^{n}\left[\left(\mu_{i}+c\sigma_{i}\right)-\left(\mu_{i}-c\sigma_{i}\right)\right]$$

where $\mu_{i}$ and $\sigma_{i}$ are the GPR predicted mean and standard deviation for each input i, c is the confidence coefficient (e.g. 1.96 for a 95 % interval), and **1{⋅}** is the indicator function (equal to 1 if the observed value lies within the prediction interval and 0 otherwise). PICP measures the proportion of observed values that fall within the model’s 95 % confidence intervals, whereas MPIW quantifies the average width of these intervals, providing a measure of interval sharpness.

Table 7, Table 8 present the values of the *R*^2^, MSE, MAPE, PICP and MPIW scores for all knot points for the distributed lateral load ($\bar{p}$) and distributed moment ($\bar{m}$) soil for the representative clay (Cowden till) and sand (Dunkirk sand) sites, respectively. The GPR models achieve consistently high *R*^2^ scores and low MSE and MAPE values, for both soil types, indicating a close match between model predictions and observed values. The PICP values are close to the nominal 95 % level and the MPIW values remain moderate, indicating that the confidence intervals are both well calibrated and sufficiently narrow. It is also noted that MPIW naturally varies between knot points, reflecting differences in the magnitude of the knot points.

**Table 7:** $R^{2}$, MSE, MAPE, PICP and MPIW metrics for the GPR models as applied to the knot point data for the distributed lateral load $\bar{p}$ and the distributed moment $\bar{m}$ related with the representative offshore glacial clay till sites.

Scores	${\bar{y}}_{1}$	${\bar{y}}_{2}$	${\bar{y}}_{3}$	${\bar{y}}_{4}$	${\bar{y}}_{5}$	${\bar{y}}_{6}$	${\bar{y}}_{7}$	${\bar{y}}_{8}$
$\bar{p}$ train data	R^2^=0.949	R^2^=0.939	R^2^=0.944	R^2^=0.969	R^2^=0.968	R^2^=0.959	R^2^=0.949	R^2^=0.941
	MSE=3.6e-4	MSE=1.4e-3	MSE=0.036	MSE=0.036	MSE=0.072	MSE=0.15	MSE=0.21	MSE=0.24
	MAPE=5.46	MAPE=4.87	MAPE=5.81	MAPE=3.41	MAPE=3.82	MAPE=5.57	MAPE=6.52	MAPE=6.97
	PICP=95.9	PICP=95.7	PICP=95.1	PICP=96.0	PICP=95.5	PICP=93.9	PICP=94.7	PICP=95.0
	MPIW=0.086	MPIW=0.174	MPIW=0.851	MPIW=0.895	MPIW=1.27	MPIW=1.77	MPIW=2.06	MPIW=2.18

$\bar{p}$ test data	R^2^=0.919	R^2^=0.927	R^2^=0.939	R^2^=0.959	R^2^=0.948	R^2^=0.936	R^2^=0.926	R^2^=0.922
	MSE=1.2e-3	MSE=3.3e-3	MSE=0.035	MSE=0.048	MSE=0.119	MSE=0.228	MSE=0.285	MSE=0.305
	MAPE=7.9	MAPE=6.8	MAPE=6.1	MAPE=4.6	MAPE=5.6	MAPE=7.7	MAPE=8.5	MAPE=8.9
	PICP=91.3	PICP=92.5	PICP=93.7	PICP=94.1	PICP=92.3	PICP=90.9	PICP=91.5	PICP=92.0
	MPIW=0.089	MPIW=0.179	MPIW=0.865	MPIW=0.919	MPIW=1.30	MPIW=1.80	MPIW=2.09	MPIW=2.21

$\bar{m}$ train data	R^2^=0.941	R^2^=0.960	R^2^=0.982	R^2^=0.967	R^2^=0.970	R^2^=0.955	R^2^=0.955	R^2^=0.956
	MSE=2.4e-5	MSE=5.3e-5	MSE=2.0e-4	MSE=4.2e-4	MSE=2.1e-4	MSE=4.6e-4	MSE=4.6e-4	MSE=4.6e-4
	MAPE=19.7	MAPE=17.9	MAPE=10.8	MAPE=6.4	MAPE=3.9	MAPE=4.6	MAPE=4.5	MAPE=4.7
	PICP=95.5	PICP=96.1	PICP=95.4	PICP=96.1	PICP=98.0	PICP=96.1	PICP=96.3	PICP=96.3
	MPIW=0.021	MPIW=0.032	MPIW=0.065	MPIW=0.093	MPIW=0.079	MPIW=0.104	MPIW=0.104	MPIW=0.104

$\bar{m}$ test data	R^2^=0.918	R^2^=0.950	R^2^=0.978	R^2^=0.943	R^2^=0.915	R^2^=0.926	R^2^=0.927	R^2^=0.927
	MSE=2.1e-5	MSE=5.3e-5	MSE=2.8e-4	MSE=6.5e-4	MSE=5.9e-4	MSE=8.1e-4	MSE=8.1e-4	MSE=8.1e-4
	MAPE=17.2	MAPE=15.7	MAPE=11.7	MAPE=7.6	MAPE=7.0	MAPE=5.6	MAPE=5.4	MAPE=5.4
	PICP=95.0	PICP=96.7	PICP=93.7	PICP=92.4	PICP=94.7	PICP=92.8	PICP=92.9	PICP=92.9
	MPIW=0.021	MPIW=0.033	MPIW=0.067	MPIW=0.095	MPIW=0.093	MPIW=0.108	MPIW=0.108	MPIW=0.109

**Table 8:** $R^{2}$, MSE, MAPE, PICP and MPIW metrics for the GPR models as applied to the knot point data for the distributed lateral load $\bar{p}$ and the distributed moment $\bar{m}$ related with the PISA representative offshore homogenous sand sites.

Scores	${\bar{y}}_{1}$	${\bar{y}}_{2}$	${\bar{y}}_{3}$	${\bar{y}}_{4}$	${\bar{y}}_{5}$	${\bar{y}}_{6}$	${\bar{y}}_{7}$	${\bar{y}}_{8}$
$\bar{p}$ train data	R^2^=0.992	R^2^=0.997	R^2^=0.999	R^2^=0.999	R^2^=0.997	R^2^=0.997	R^2^=0.989	R^2^=0.977
	MSE=4.2e-5	MSE=3.9e-4	MSE=1.9e-3	MSE=5.9e-3	MSE=0.019	MSE=0.13	MSE=0.65	MSE=1.41
	MAPE=10.8	MAPE=3.99	MAPE=1.60	MAPE=1.37	MAPE=1.44	MAPE=2.65	MAPE=5.17	MAPE=6.65
	PICP=96.3	PICP=95.7	PICP=97.5	PICP=97.5	PICP=98.0	PICP=97.4	PICP=96.7	PICP=96.6
	MPIW=0.032	MPIW=0.091	MPIW=0.236	MPIW=0.458	MPIW=0.885	MPIW=2.20	MPIW=4.34	ARIL=6.12

$\bar{p}$ test data	R^2^=0.988	R^2^=0.996	R^2^=0.999	R^2^=0.998	R^2^=0.997	R^2^=0.995	R^2^=0.982	R^2^=0.965
	MSE=4.4e-5	MSE=3.6e-4	MSE=2.6e-3	MSE=0.020	MSE=0.043	MSE=0.21	MSE=1.06	MSE=2.17
	MAPE=10.5	MAPE=5.08	MAPE=1.92	MAPE=2.17	MAPE=2.38	MAPE=4.19	MAPE=6.62	MAPE=8.11
	PICP=95.7	PICP=95.1	PICP=95.7	PICP=94.4	PICP=94.4	PICP=96.9	PICP=96.5	PICP=96.3
	MPIW=0.032	MPIW=0.097	MPIW=0.258	MPIW=0.522	MPIW=1.06	MPIW=2.55	ARIL=4.64	MPIW=6.41

$\bar{m}$ train data	R^2^=0.970	R^2^=0.948	R^2^=0.999	R^2^=0.998	R^2^=0.998	R^2^=0.992	R^2^=0.989	R^2^=0.975
	MSE=4.6e-6	MSE=1.6e-4	MSE=2.2e-5	MSE=3.5e-4	MSE=2.3e-3	MSE=0.021	MSE=0.034	MSE=0.071
	MAPE=12.6	MAPE=11.0	MAPE=0.94	MAPE=2.72	MAPE=5.60	MAPE=8.44	MAPE=10.2	MAPE=11.5
	PICP=99.4	PICP=95.1	PICP=98.6	PICP=98.5	PICP=97.1	PICP=97.1	PICP=97.1	PICP=96.3
	MPIW=9.4e-3	MPIW=0.055	MPIW=0.031	MPIW=0.110	MPIW=0.259	MPIW=0.790	MPIW=1.01	MPIW=1.38

$\bar{m}$test data	R^2^=0.961	R^2^=0.938	R^2^=0.998	R^2^=0.997	R^2^=0.997	R^2^=0.989	R^2^=0.981	R^2^=0.963
	MSE=3.3e-6	MSE=1.5e-4	MSE=5.8e-5	MSE=1.2e-3	MSE=3.8e-3	MSE=0.026	MSE=0.047	MSE=0.11
	MAPE=13.6	MAPE=13.9	MAPE=1.83	MAPE=3.56	MAPE=7.01	MAPE=12.7	MAPE=12.9	MAPE=14.1
	PICP=98.8	PICP=94.6	PICP=94.5	PICP=95.1	PICP=94.5	PICP=95.8	PICP=98.1	PICP=96.1
	MPIW=9.5e-3	MPIW=0.056	MPIW=0.039	MPIW=0.126	MPIW=0.278	MPIW=0.845	MPIW=1.09	MPIW=1.46

The GPR models are used to predict the knot point data required for reconstructing the soil reaction curves. Specifically, the models generate depth-dependent knot point values for the distributed lateral load (​$\bar{p}$) and distributed moment ($\bar{m}$), as well as the knot point prediction values for the base horizontal force (${\bar{H}}_{B}$​) and base moment $({\bar{M}}_{B}$). To demonstrate the performance of the GPR approach to predict the knot point data, data relating to Piles C10 and C11 from Table 3, embedded in a clay soil profile with $d_{ice}$ = 25 m and in a sand profile with $D_{R}$ = 75 %, have been arbitrarily selected. The variation with depth of the (${\bar{y}}_{1}$, ${\bar{y}}_{4}$, ${\bar{y}}_{7}$) knot point parameters along the pile for distributed lateral load ($\bar{p}$) and distributed moment ($\bar{m}$) for this case are plotted in Fig. 8, Fig. 8.

**Fig. 7:**
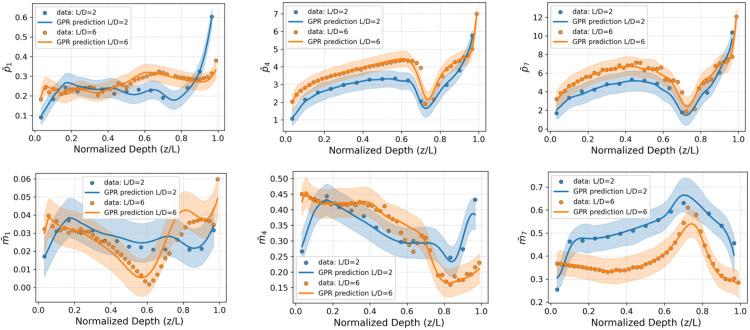
Illustration of the performance of the GPR models for knot point values (${\bar{y}}_{1}$, ${\bar{y}}_{4}$, ${\bar{y}}_{7}$) of the distributed lateral load $\bar{p}$ (top row) and distributed moment $\bar{m}$ (bottom row). The data correspond to the representative offshore clay site with $d_{ice}$ = 25 m, as presented in Fig. 5, for piles C10 and C11. Data indicated by blue and orange circles for L/D=2 and L/D=6, respectively, represent the corresponding knot point values of the soil reaction curves extracted from the finite element calibration analyses.

**Fig. 8:**
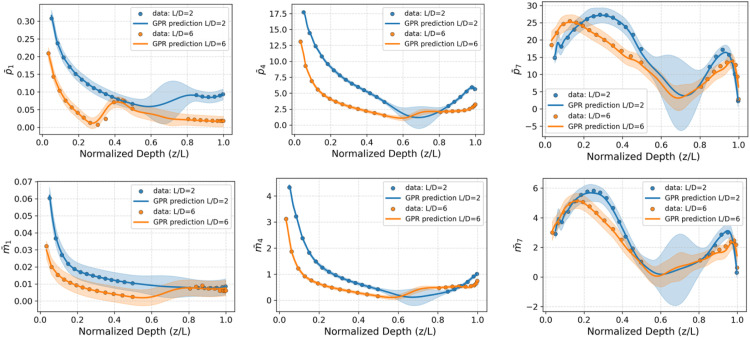
Illustration of the performance of the GPR models for knot point values (${\bar{y}}_{1}$, ${\bar{y}}_{4}$, ${\bar{y}}_{7}$) of the distributed lateral load $\bar{p}$ (top row) and distributed moment $\bar{m}$ (bottom row). The data correspond to the representative offshore sand site with $D_{R}$ of 75 %, as presented in Fig. 6, for piles C10 and C11. Data indicated by blue and orange circles for L/D=2 and L/D=6, respectively, represent the corresponding knot point values of the soil reaction curves extracted from the finite element calibration analyses.

Fig. 7, Fig. 8 indicate that the GPR models reproduces the nonlinear depth-dependent behaviour that is evident in the numerical calibration data with high accuracy. Similar performance is observed for the other cases as indicated by the accuracy and confidence interval metrics in Table 7, Table 8. The shaded regions in the figures denote the 95 % confidence intervals, illustrating the built-in uncertainty quantification capability of the GPR model. In the sand model, the confidence intervals widen near the pile’s centre of rotation due to the absence of data in this region – an issue arising from difficulties in extracting reliable values from 3D finite element calibration analyses [2]. In contrast, the clay model includes data near the rotation point, resulting in consistent confidence intervals along the full pile depth.

It should be noted that the corresponding mobilised soil reaction curves near the pile’s rotation point – specifically, the non-dimensional distributed lateral load, $\bar{p}$, in this region – are relatively small [2] and have a negligible influence on the overall pile lateral response. Hence, relatively large prediction uncertainties near the rotation point have a negligible effect on the accuracy of the overall 1D model.

### Alternative machine learning model candidates

The proposed data-driven 1D model framework is not limited to the use of GPR to determine the soil reaction curves; other regression-based machine learning models could be employed. Potential candidate models include artificial neural networks (ANNs) and extreme gradient boosting (XGBoost). These alternative models could be beneficial for very large calibration datasets as in such cases the GPR model can become computationally demanding due to its non-parametric nature, which results in high training time and memory requirements.

Although a detailed study of these alternative models is outside the scope of the current paper, Table 9 presents, for reference, the average *R^2^* scores (training and testing) and the corresponding training times obtained for the ANN and XGBoost models using the databases of the general sand and clay models employed in this paper. The optimal hyperparameter sets for each knot point parameter were determined using randomised search over a wide range of possible parameter values. The results indicate that both ANN and XGB models could be effective machine learning candidates for the data-driven 1D model.

**Table 9:** Average $R^{2}$ values for the GPR, ANN and XGBoost models applied to the knot point data for the distributed lateral load $\left(\bar{p}\right)$ and the distributed moment ($\bar{m}$). For the ANN, a feedforward neural network was applied with two or three hidden layers was used. The reported training times for the ANN and XGBoost models exclude the hyperparameter tuning process.

ML Model	Cowden till					Dunkirk sand				
	Time	$\bar{p}$ (train)	$\bar{p}$ (test)	$\bar{m}$ (train)	$\bar{m}$ (test)	time	$\bar{p}$ (train)	$\bar{p}$ (test)	$\bar{m}$ (train)	$\bar{m}$ (test)
GPR	15min	0.962	0.945	0.952	0.931	11min	0.975	0.973	0.963	0.962
ANN	2min	0.949	0.931	0.941	0.926	2min	0.957	0.945	0.950	0.935
XGBoost	1min	0.944	0.933	0.938	0.924	1min	0.952	0.942	0.945	0.930

### Incorporation of the machine learning predictions in the 1D model framework

**Fig. 9:**
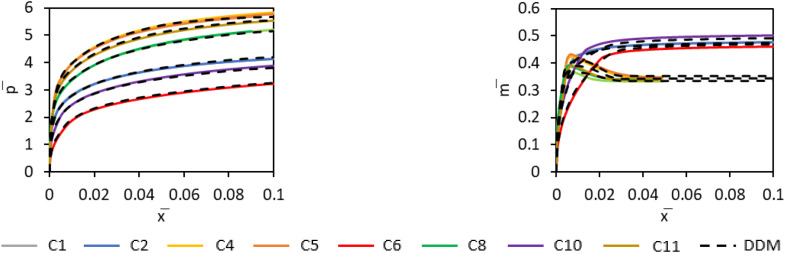
Normalised numerical distributed soil reaction curves $\bar{p}$, $\bar{m}$ at a representative depth below the ground level z/L=0.225 for piles C1 to C11 embedded in the representative offshore glacial till site with $d_{ice}=50m$. Results are shown as calculated from PLAXIS 3D finite element analyses and from the data-driven 1D design model (referred to as DDM). C3 and C7 piles form nearly identical soil reaction curves with piles C1 and C6 respectively and therefore are omitted from the graphs.

The GPR knot point predictions are converted into continuous soil reaction curves using the PCHIP interpolation scheme; the spline functions can be readily embedded within the 1D finite element model that is employed to compute the overall performance of the monopile. To assess the performance of the data-driven framework in predicting the soil reaction curves, a study was conducted on comparing the PCHIP soil reaction curves with the corresponding numerical soil reaction curves for the calibration cases. The results indicate that the PCHIP curves provide a high-fidelity match with the numerical data across the full displacement range. An illustrative comparison for the Cowden till model is shown in Fig. 9, which presents the distributed lateral load ($\bar{p}$) and distributed moment ($\bar{m}$) curves for all of the calibration piles presented in Table 3 embedded in the representative offshore glacial till site with $d_{ice}=50m$. The data-driven model predictions (black dashed lines) closely match the numerical soil curves (solid lines), including cases that exhibit post-peak softening behaviour. Although detailed data are not presented here, the sand model predictions demonstrated a similar level of accuracy, confirming the robustness of the data-driven model for different soil types.

**Fig. 10:**
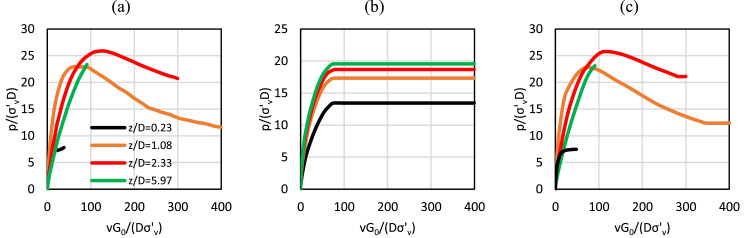
Normalised distributed lateral load, $\bar{p}$, for pile C4 embedded in sand with $D_{R}$=75 %*.* Data are shown for normalised depths z/D=0.23, 1.08, 2.33, 5.9: (a) numerical soil reaction curves. (Data equivalent to Fig. 8a of [5]); (b) PISA conic function (stage 1 optimisation) representation of the soil reaction curves. (Data equivalent to Fig. 8a of [5]); and (c) soil reaction curves determined using the data-driven model developed in this study, employing GPR. For the purpose of this comparison, the dimensionless form $vG_{0}/D\sigma_{v}^{\prime }$ is adopted in Fig (c) for consistency with the dimensionless forms adopted in the PISA design model.

The eight-point PCHIP spline formulation employed in the current work provides an improved representation of the soil reaction curves, compared with the conic function employed in the PISA design model, as illustrated in the data in Fig. 10 for example data on normalised distributed lateral load ($\bar{p}$). Fig. 10a shows the numerical soil reaction curves for the example case; Fig. 10b shows the corresponding soil reaction curves obtained from the stage one optimisation process in the calibration of the PISA design model. The data in Figs. 10a and b are taken directly from Fig. 8 of Burd et al. [5]. It is clear that the conic function employed in the PISA design model is unable to model the full detail of the numerical soil reaction curves. The PCHIP spline function approach – as adopted in the current work – is plotted in Fig 10c; this provides a very good match with the numerical data.

**Fig. 11:**
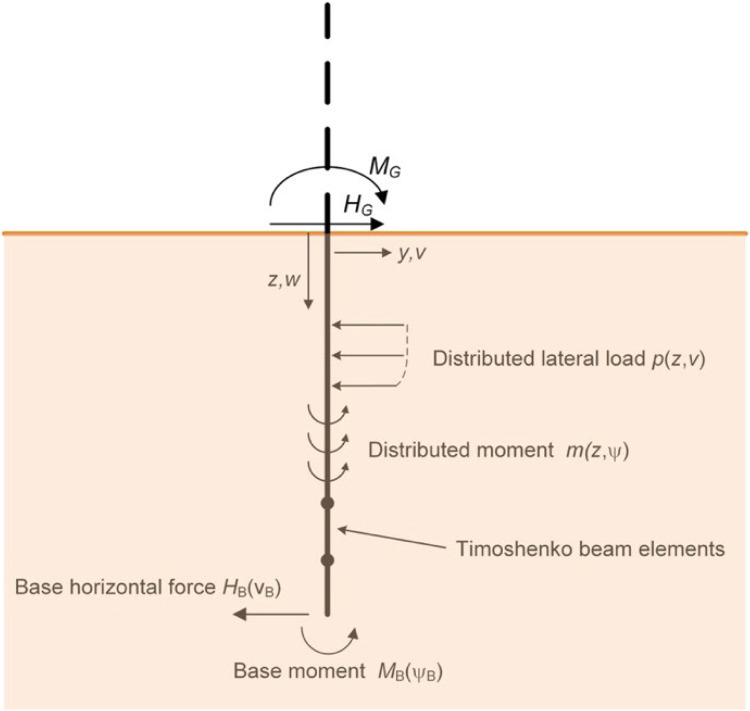
1D finite element implementation of the data-driven design model. (Adapted from Byrne et al. [9] and [5]).

The PCHIP soil reaction curves are integrated into a 1D nonlinear finite element implementation – incorporating a beam representation of the embedded monopile – as illustrated in Fig. 11 to compute the monotonic lateral response of the monopile. This includes predictions of ground-level load-displacement and moment-rotation responses – which are typically used to define the monotonic loading backbone curve – and the distribution of bending moment and shear force along the embedded length of the pile. The underlying 1D finite element implementation, employing Timoshenko beam theory and consistent 1D elements to represent the distributed soil reactions, follows the formulation detailed in Byrne et al. [9] and Burd et al. [5] and is not discussed in detail here.

## Method validation

**Fig. 12:**
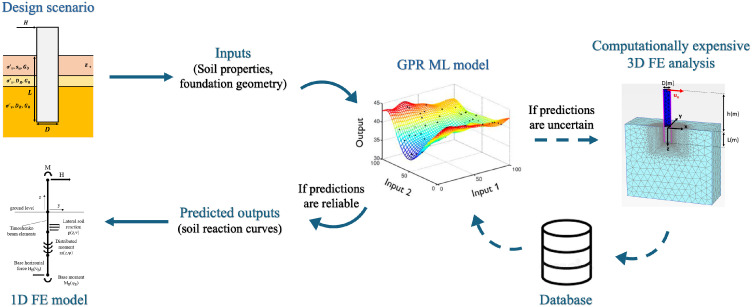
Schematic flowchart indicating the key stages to use a data-driven 1D design model for design calculations.

The data-driven 1D design model developed in the current study is intended for the analysis of offshore wind turbine monopiles with geometries and soil conditions that differ from those used in the calibration analyses. The general workflow for applying the models in design calculations is illustrated in Fig. 12. It is recommended that the model is only used for analyses within the calibration space, where the GPR models can reliably predict soil reaction curves for unseen inputs. If the model is used for calculations employing pile geometries or soil profiles that fall outside the calibration space, caution is required. In such cases, a validation analyses should be conducted to confirm the robustness of the results.

Two representative design scenarios are employed to demonstrate the capabilities of the proposed model, as follows:

• Design monopiles in homogeneous soil profiles with unseen strength and stiffness properties and unseen pile geometry. The clay model is evaluated for three unseen glacial till sites with ice depths of $d_{ice}=15,\;40,\;75$m, while the sand model is tested for unseen sites with relative densities of 55 %, 70 % and 85 %.
• Design monopiles in a layered soil profile – where each layer adopts the corresponding homogeneous soil calibration.

Two design pile configurations, D1 and D2t (as defined in [9], and [5]), are employed in the analyses as specified in Table 10.

**Table 10:** Design pile geometries.

Pile	D:m	h:m	h/D	L:m	L/D	t:mm	D/t
D1	7.5	37.5	5	22.5	3	68	110
D2t	8.75	87.5	10	35	4	150	58

### Uncertainty estimation of GPR predictions in design scenarios

GPR models are used to predict the knot point data for unseen inputs. The GPR approach naturally provides 95 % confidence interval widths, w, on the model predictions for individual knot points (e.g. as shown in Fig. 7, Fig. 8). While these confidence interval widths indicate the level of confidence in the prediction of individual knot point data, it is not straightforward to use these confidence intervals to quantify the reliability of calculations on the overall performance of a monopile conducted using the 1D model. However, it is possible to gain an indirect indication of model uncertainty via the uncertainty measure, $U_{M}$ which is defined:

(8) $$U_{M}=w\left(x_{*}\right)/{\bar{w}}_{train}$$

where $w(x_{*})$ is the width of the 95 % confidence interval at a specific knot point for the unseen inputs $x_{*}$ and ${\bar{w}}_{train}$ is the mean of the 95 % confidence interval for the specific knot point for the complete set of training data. The value of $U_{M}$ provides an indication of the reliability of the prediction; high values of $U_{M}$ signify an unreliable prediction. To obtain a measure of uncertainty for entire monopile analysis, the value of $U_{M}$ is averaged over all of the knot points in the problem; the resulting parameters is termed averaged model uncertainty, ${\bar{U}}_{M}$. Based on the authors’ experience, a threshold of ${\bar{U}}_{M}=3$ was found to perform well for both the clay and sand models presented in this study. However, this threshold value depends on the density and distribution of the training data and may needs adjustment for future model developments.

An alternative approach to assess model uncertainty is to determine the Mahalanobis distance, $D_{M}$ [12,19], which is defined:

(9) $$D_{M}=\sqrt{{\left(x_{*}-\mu_{x}\right)}^{T}{\Sigma_{x}}^{-1}\left(x_{*}-\mu_{x}\right)}$$

where $x_{*}$ is an unseen input vector**,** $\mu_{x}$ is the mean vector of the training data and $\Sigma_{x}$ is the covariance matrix of the training data. The Mahalanobis distance provides a multivariate measure of dissimilarity between an unseen input sample and the training data. An overall measure of reliability can be obtained by averaging the Mahalanobis distances ($D_{M}$) across all unseen input samples to yield a mean value (${\bar{D}}_{M}$). Small ${\bar{D}}_{M}$ values indicate that the inputs are similar to the training data and that the GPR predictions are reliable. Large ${\bar{D}}_{M}$ values suggest predictions may not be trustworthy. To determine threshold values of $D_{M}$, the empirical 97.5th percentile of the training distribution of Mahalanobis distances, computed after standardisation, can be adopted. This corresponds to $D_{M}=3.07$ for the clay model and $D_{M}=2.79$ for the sand model. These threshold values are also considered applicable to the mean value of the Mahalanobis distance ${\bar{D}}_{M}$.

Values of ${\bar{U}}_{M}$ and ${\bar{D}}_{M}$ provide a practical means of assessing the reliability of GPR predictions. In cases where the predictions are deemed uncertain – based on values of the metrics that exceed pre-defined thresholds – a 3D FE analysis should be conducted to check the 1D model predictions.

Table 11 lists the corresponding ${\bar{U}}_{M}$ and ${\bar{D}}_{M}\;$ values for all design scenarios involving monopiles embedded in homogeneous soil profiles with unseen strength and stiffness properties and unseen pile geometry. The values of ${\bar{U}}_{M}$ and ${\bar{D}}_{M}$ are all below the suggest thresholds confirming the reliability and safety of the GPR-predicted knot point data. These predictions can therefore be confidently employed within the 1D finite element framework to evaluate the monotonic lateral response of the pile.

**Table 11:** Average uncertainty measure (${\bar{U}}_{M}$) values for all predicted knot points and average Mahalanobis distance (${\bar{D}}_{M}$) values for all inputs determined for the design monopiles embedded in homogeneous soil profiles with unseen strength and stiffness properties and unseen pile geometry.

Metrics	D2t - $D_{R}$=55 %	D2t - $D_{R}$=70 %	D2t - $D_{R}$=85 %	D1 - $d_{ice}$=15m	D1 - $d_{ice}$=40m	D1 - $d_{ice}$=75m
$U_{M}$	2.24	2.16	2.15	1.52	1.18	1.16
$D_{M}$	1.76	1.58	1.93	2.18	2.04	2.03

### Predictions for design monopiles embedded in homogenous soil profiles

**Fig. 13:**
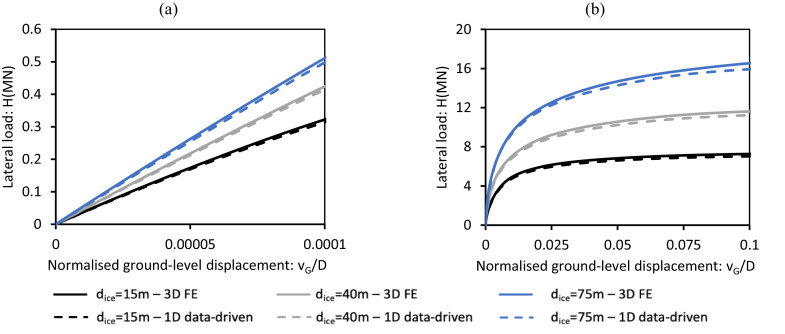
Comparisons between the 3D finite element validation analyses (indicated as ‘3D FE’ and the Cowden till data-driven 1D design model (indicated as ‘1D data-driven’ for design pile D1 for glacial till sites with $d_{ice}=15,\;40,\;75m$: (a) small displacement response, (b) ultimate response. The 3D FE data are calculated following the procedure detailed in Kamas et al. [16].

Fig. 13 presents the lateral load vs. ground-level pile displacement response for small ($v_{G}=D/10000$) and ultimate ($v_{G}=D/10$) reference displacements computed by the general Cowden till data-driven 1D design model for pile D1 embedded in three unseen representative offshore glacial till sites corresponding $d_{ice}=15,\;40,\;75m$. Results from corresponding 3D finite element ‘validation’ analyses conducted using PLAXIS 3D are also shown. The data-driven 1D model provides a close match to the FE results for both small and large displacement responses, demonstrating that the 1D model can accurately reproduce the monopile lateral response under these conditions.

**Fig. 14:**
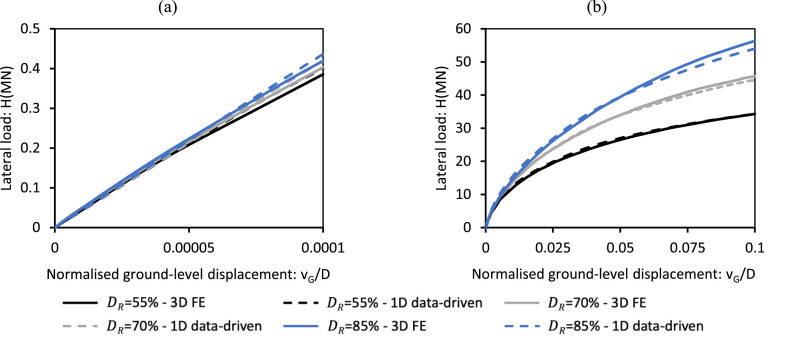
Lateral load vs. ground-level pile displacement data determined using the sand data-driven 1D design model (indicated as ‘1D data-driven’) for design pile D2t for sand with relative densities 55 %, 70 % and 85 %. Also shown (indicated as ‘3D FE’) are data from corresponding 3D finite element validation analyses: (a) small displacement response, (b) ultimate response. The 3D finite element analyses are described in Burd et al. [5]; ‘3D FE’ data are identical to those plotted in Fig. 15 of Burd et al. [5].

A similar comparison has been conducted for the data-driven 1D design model for sand employing pile D2t. Fig. 14 shows the pile’s load-displacement response at ground level computed by the data-driven model and corresponding 3D finite element validation analyses (conducted using ICFEP as described in [5]) for small and ultimate reference displacements for pile D2t embedded in unseen sand sites with relative densities 55 %, 70 % and 85 %. The data-driven model closely aligns with the validation analyses in all cases.

**Fig. 15:**
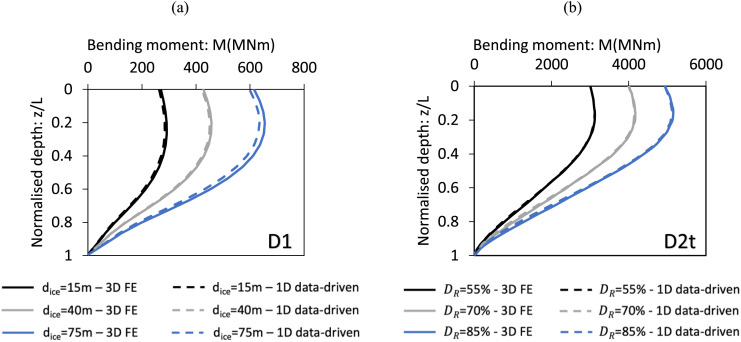
Bending moments profile below ground level determined using (a) the general Cowden till data-driven 1D model for pile D1, (b) the general Dunkirk sand data-driven 1D model for pile D2t. Also shown are data from the corresponding 3D finite element validation analyses. The bending moments shown are determined for a value of horizontal load $H=H_{ult}$ where $H_{ult}$ is ultimate lateral load determined from the validation analyses at a ground-level displacement of $v_{G}=0.1D$. ‘3D FE’ data in Fig. a are calculated by PLAXIS 3D following the procedure detailed in Kamas et al. [16]; ‘3D FE’ data in Fig. b for $D_{R}=55\%$ and $D_{R}=85\%$ are identical to those plotted in Fig. 15 of Burd et al. [5]; data for $D_{R}=70\%$ are taken from AWG [3].

Fig. 15 presents a comparison of below-ground bending moments for the design scenarios presented in Fig. 13, Fig. 14, as determined by the data-driven 1D design models and corresponding 3D finite element analyses. The results demonstrate a close match between the 1D and 3D models.

### Accuracy and ratio metrics

**Fig. 16:**
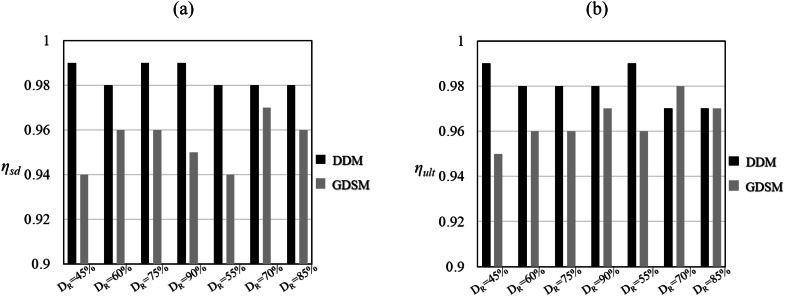
Accuracy (η) metrics calculated for the data-driven 1D model (DDM) for sand and the GDSM PISA design model for: (a) the small displacement, and (b) ultimate response cases. The results for the GDSM PISA design model have been directly taken from Table 8 of Burd et al. [5].

Values of accuracy (η) metrics (defined in [9]) are used to quantify the accuracy of the data-driven 1D design model calculations for an analysis set that includes the 11 calibration piles in Table 3 and the design piles in Table 10. The η metric is defined (see Fig. 6 of [9]) as:

$\eta =1-\frac{A_{diff}}{A_{ref}}$ (6) where $A_{ref}$ is the area below the H-$v_{G}$ curve computed using the 3D model (for a specified range of $v_{G}$); $A_{diff}$ is the corresponding area indicating the difference between theH-$v_{G}$ curve computed using the 3D model with theH-$v_{G}$ curve computed using the 1D model. Average accuracy metrics for the sand profiles are shown in Fig. 16a and b, respectively. Consistent with the nomenclature in Byrne et al. [9], $\eta_{sd}$ relate to small displacements ($v_{G}\leq D/10000$) and $\eta_{ult}$ relate to ultimate response ($v_{G}\leq D/10$). Since all η metric values are close to unity, the data-driven model is seen to provide an accurate representation of the corresponding 3D finite element analyses. For reference, the accuracy metrics determined from the Generalised Dunkirk sand (GDSM) PISA design model by Burd et al. [5] are also included, for the same soil reaction curve database. It can be seen that the data-driven model achieves higher values of accuracy metric than the PISA design model in all cases for the small displacement metric and the majority of cases for the ultimate response metric. A similar pattern of behaviour is observed for the relative performance of the PISA design model and the data-driven model for clay soils.

### Layered soil analyses

Although the data-driven model developed in the current work is calibrated for homogeneous soil profiles (i.e. either glacial till or sand), the method may be applied straightforwardly to layered soils by incorporating the appropriate (homogeneous) calibration for each of the soil layers. In this approach (termed ‘independent layer method’) potential interactions between the layers are absent from the model. Nevertheless – consistent with the findings in Burd et al. [6] in connection with the PISA model – the data-driven model employing the independent layer method is found to provide accurate predictions of the performance of a monopile in layered soils (provided that the strength and stiffness of adjacent soil layers are not too dissimilar).

**Fig. 17:**
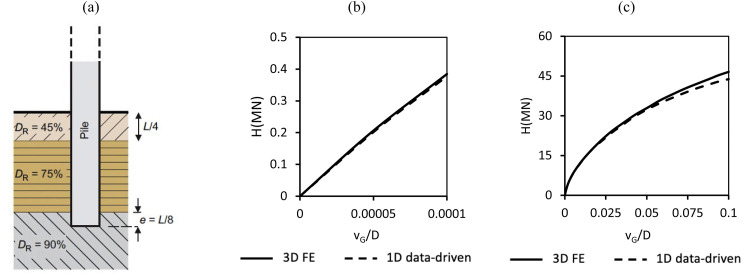
(a) Case E1A configuration [6]. Comparisons between the 3D finite element validation analysis conducted using ICFEP, AWG [3] and the data-driven 1D model for pile D2t for: (b) small displacement response, (c) ultimate response.

**Fig. 18:**
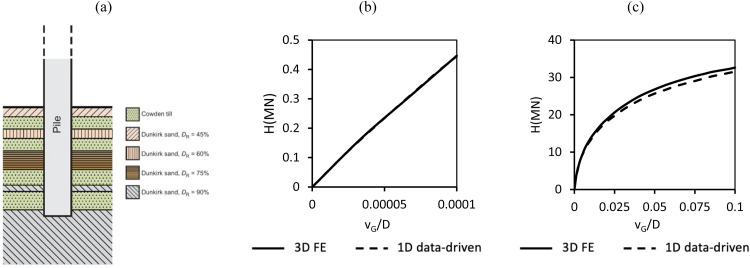
(a) Case E3 configuration [6]. Comparisons between the 3D finite element validation analysis conducted using ICFEP, AWG [3] and the data-driven 1D model for pile D2t for: (b) small displacement response, (c) ultimate response.

To demonstrate the approach, analyses have been conducted for pile D2t embedded in the layered soil profiles shown in Fig. 17, Fig. 18. The profile in Fig. 17a corresponds to Case E1A in Table 9 of Burd et al. [6], while profile in Fig. 18a corresponds to Case E3 in Table 9 of Burd et al. [6]. For both layered soil profiles, the data-driven 1D design model computes accurately the load-displacement response obtained from previous 3D finite element validation analyses conducted using ICFEP [3]. In these cases, the adjacent soil layers exhibit only moderate variations in strength and stiffness – for example, sands with variable density (Case E1A) or stiff overconsolidated clay overlain or underlain by sands of differing relative density (Case E3). Consistent with the results presented in [6] – employing the PISA design model – the accuracy of the model can be significantly reduced when adjacent layers have strongly contrasting stiffness and/or strength characteristics.

### Discussion

The paper describes a data-driven model that is capable of delivering high-quality representations of the behaviour of a monopile within a specified design space. The paper demonstrates that (i) the PCHIP spline interpolation approach employing a GPR model to determine the knot point data provides a robust way of representing the soil reaction curves and (ii) the overall accuracy of the model – e.g. as indicated by numerical accuracy metrics – is consistently high for cases within the calibration space. These outcomes are confirmed by numerous other sensitivity and accuracy studies that have been conducted on this modelling approach – as described in Kamas [17].

To achieve a robust calibration of the data-driven 1D design model, suitable data from 3D finite element analyses conducted within a defined calibration space are needed. The current work adopts the pile geometries and soil profiles that were previously developed in connection with the development of the PISA design model (although additional clay profiles are incorporated in the current work). Considerable investment of time and computing resources is needed to conduct the 3D finite element calibration analysis and future work is needed to investigate efficient strategies for the specification of the calibration analyses. If too few calibration analyses are conducted – or if the calibration analyses are inefficiently distributed across the calibration space – then the fidelity of the calibrated 1D model will be reduced. It is considered that the number and distribution of the calibration analyses employed in the current work is sufficient to calibrate a 1D model that is robust within the calibration space. Preliminary investigations (described in [17]) indicate that the removal of several of the 3D finite element analysis from the calibration data has only a minimal influence on the overall performance of the 1D model. But a detailed study on the way in which adjustments to the number and spread of the calibration data influence the fidelity of the 1D model has not yet been conducted.

Importantly, the predictive accuracy of the 1D model relies heavily on the consistency and high quality of the underlying calibration data. It is therefore recommended that only high-quality calibration data are used to develop or enhance existing databases. Quality control procedures for the 3D finite element calibration calculations should follow normal good practice. To merge 3D finite element data from separate projects, care is needed (supported by documentation) to ensure that the reliability of the analyses is appropriate. All input features, soil reaction knot point data, and associated 3D FE calibration data should be stored in a structured database to allow efficient tracking, sharing, and integration of new information. Such a system would allow calibration databases to be updated with new calibration data when updating models for different pile geometries or soil conditions.

The current paper employs a GPR model to determine the knot point data for the purpose of defining the soil reaction curves in the 1D model. In the calibration procedures described in the current paper the training time was about 11 min for the sand model and 15 min for the clay model; each model calibration employed 44 3D finite element calibration analyses. While these training times are not excessive it should be noted that increasing the number of 3D finite element calibration analyses would increase the training time very considerably; this is a consequence of the non-parametric nature of the GPR model. For large training data sets, alternative machine learning methods such as Neural Networks or XGBoost might be preferable, as they offer comparable accuracy with lower training times. Alternatively, sparse GPR formulations (e.g., [17]) could be adopted; these methods use representative subsets of the training data to reduce computational demand without compromising accuracy. Once trained, however, the GPR models are computed rapidly.

The method described in the current paper is concerned with calibrating a surrogate model within a specified calibration space. Within the calibration space the model is expected to produce high fidelity predictions, but questions naturally arise on the likely performance of the model when it is used outside of its calibration space. It has been discovered – on the basis of experimentation – that the inputs in the current model can depart a small amount (typically up to about 20 %) from the calibration space without a significant degradation in performance. On a more formal basis, two methods are presented (relating to the average uncertainty metric ${\bar{U}}_{M}$ and average Mahalanobis distance ${\bar{D}}_{M}$) to quantify the likely model uncertainty for specific cases. Combinations of input parameters that lead to values of these metrics that exceed specified thresholds (e.g. relating to departures from the calibration space) indicate significant potential uncertainty. In these cases it is recommended that additional 3D finite element analyses are conducted to check the accuracy of the results.

The method described in the current paper comprises separate calibration procedures for clay and sand sites. However, layered soil profiles are typically encountered at offshore sites. The current paper describes the application of an ‘independent layer’ method for the analysis of a monopile at a layered soil site. The examples presented in the paper demonstrate accurate results using this approach. However, it should be noted that the independent layer approach represents a departure from the calibration space (on the basis that the calibration analyses were all conducted with homogeneous soils). As a consequence, the reliability of the method for layered soil analysis is uncertain. Although the two layered soil examples presented in the paper indicate that the 1D model produces accurate results, it is possible to define cases involving adjacent soil layers with highly contrasting strength and stiffness characteristics in which the fidelity of the model predictions is low. Although beyond the scope of the current paper, it is feasible to develop layered soil calibrations in which the geometry of the layering is included as additional features. Models of this sort (as described in [17]) provide high-fidelity predictions even for cases where significant differences exist between the strength and stiffness of adjacent layers.

Site-specific data can be incorporated straightforwardly within the calibrated model, provided that the geotechnical parameters fall within the calibration bounds. For novel site conditions (e.g. employing chalk or clay-sand mixtures) in which the geotechnical behaviour differs from the calibration cases then additional 3D finite element calibration analyses would be required to develop a new calibration.

## Limitations

The data-driven 1D design model presented in the current work is a form of surrogate model that exhibits strong predictive performance and scalability. However, the following limitations should be acknowledged.

The reliance on machine learning methods in the proposed model introduces a significant threshold for practical applications of the method for design purposes. In contrast to the PISA design model, which employs simple functions for the soil reaction curves with a relatively small number of parameters that can conveniently be presented in human-readable form, the current data-driven approach operates as a ‘black-box’. Employing black-box design procedures of the sort described in the current paper represents a departure from conventional design calculation procedures. The calibration data (comprising the results obtained from 3D finite element calibration analyses) are, of course, highly portable in electronic form; it therefore seems feasible to develop practical design procedures – based on the current approach – in which design calculations are conducted on the basis of (portable) calibration data sets that have been suitably quality assured. However, it is acknowledged that this approach does not have the level of transparency and interpretability that is a characteristic of models (such as the PISA design model) for which the calibration parameters and functions can be conveniently represented in tabular form.

The proposed method relies on the generation of a significant amount of training data; this process is potentially costly in terms of time and computational resources. Although the initial 3D FE calibration phase requires a significant time investment, subsequent design iterations with the calibrated 1D model can be completed rapidly. There is therefore a trade-off between a significant investment of time in the calibration phase which must be balanced with the high efficiency of the trained 1D model. It is acknowledged, therefore that a significant investment is needed to develop model calibrations for novel cases (e.g. involving new soil types such as clay-sand mixtures or chalk) and that the need for this investment may limit the applicability of the method in practical design workflows.

Although the 3D analyses that are employed to calibrate the proposed model can be conducted using commercial software (for example, PLAXIS 3D), bespoke software tools are required: (i) to extract the soil reaction curves; (ii) to implement the GPR model (for the knot point data); and (iii) to implement the 1D finite element model. The fact that the complete workflow cannot be executed solely within currently-available commercial software presents a significant threshold that needs to be overcome before the method can be used for engineering design applications.

The models described in the paper are calibrated for homogeneous soil conditions. While it is possible to achieve reliable predictions for layered soil cases, this represents an application of the model that is outside of the calibration space. The reliability of the model when applied to layered soils cannot be assured, especially in cases where adjacent soil layers have strongly contrasting strength and stiffness characteristics. However, this limitation can be overcome by employing a 1D model that is directly calibrated for layered soil cases, with the geometry of the layers being specified as features. A detailed presentation on direct calibration procedures for layered soils is, however, outside of the scope of the current paper.

The current model is limited to monotonic loading conditions. While the framework could, in principle, provide elastic stiffness inputs for global dynamic analyses of wind-turbine support structures, it does not capture cyclic or time-dependent soil behaviour

## Ethics statements

This work did not involve human subjects, animal experiments data, and data collected from social media platforms.

## CRediT author statement

**Ioannis Kamas**: Conceptualization, Investigation, Formal analysis, Methodology, Software, Writing – original draft. **Stephen K. Suryasentana**: Conceptualization, Investigation, Methodology, Writing – review & editing. **Harvey Burd**: Conceptualization, Supervision, Writing – original draft. **Byron Byrne**: Conceptualization, Supervision, Funding acquisition, Writing – review & editing.

## Declaration of interests

The authors declare that they have no known competing financial interests or personal relationships that could have appeared to influence the work reported in this paper.

## Data Availability

Data will be made available on request.
